# Thank you to our peer reviewers in 2023

**DOI:** 10.1111/ene.16213

**Published:** 2024-01-22

**Didier Leys** (*Editor‐in‐Chief*), **Claudia Sommer** (*Deputy‐Editor*), **Barbara Borroni**, **Andrew Chan**, **Mark Edwards**, **Alessandro Tessitore**, **Kristl Vonck** (*Associate Editors*), **Paulo Caramelli**, **Isabel Martins**, and **Radu Tanasescu** (*Guest Editor*s).

Would like to thank all the reviewers from around the world who have provided expert advice to ensure publication of the best quality articles in the *European Journal of Neurology*, the journal of the *European Academy of Neurology*. Their feedback is crucial to improve the quality of manuscripts and of neurological care provided to patients. The names of those who reviewed manuscripts for the *European Journal of Neurology* in 2023 are listed below. Our warmest gratitude goes to all of them.

*Indicates those who reviewed 5 to 9 manuscripts in 2023, ** indicates those who reviewed 10 to 14, and *** indicates those who reviewed 15 or more (reviews of revised manuscripts are not counted).

Abdelhalk Ahmed

Abresch Richard

Abu‐Rumeileh Samir

Adamec Ivan

Adams David

Adhikary Asit Baran

Agarwal Apeksha

Agosta Federica

Aguglia Umberto

Aguiar De Sousa Diana

Ahmed Rebekah

Akushevich Igor

Alam Uazman

Alamowitch Sonia

Albert George

Alboini Paolo

Alhusaini Saud

Allen Jeffrey A.*

Allen‐Philbey Kimberley

Almekhlafi Mohammed

Almendra Ricardo

Alonso‐Cánovas A.

Alonso‐Jimenez Alicia

Altmann Patrick

Altomare Daniele

Alvarez Jorge I.

Alves Luisa

Alves Pedro

Amarenco G.

Amaro Sergio

Amboni Marianna

Andersen Henning*

Anderson Craig

Andrews P. Ian

Angelini Corrado

Anzai Yoshimi

Appleby Brian

Aprile Vittorio

Arantes Paula M. M.

Araujo Abelardo

Arba Francesco

Areskeviciute Ausrine

Arsava Ethem

Arsian Seckin

Arya Ravindra

Ashina Hakan

Ashina Messoud

Ashkenazi Avraham

Asken Breton

Aslaksen Per

Astorino Todd A.

Atkins Hl

Attarian Shahram

Attarian Shahram

Aubinet Charlène

Audebert Heinrich

Auer‐Grumbach Michaela

Axer Hubertus

Aybek Selma

Bachoud‐Lévi Anne‐Catherine

Bahra Anish

Baillieul Sebastien

Baker Susanne

Balabanski Anna

Baldelli Luca

Banerjee Gargi

Barbanti Piero

Barisano Giuseppe

Barloese Mads

Barnett Carolina

Barsottini Og

Bartsch Peter

Battini Roberta

Baxendale S.

Baykan Betül

Becker Jefferson

Bede Peter

Beier Christoph*

Beier Christoph Patrick

Beier Dagmar

Béjot Yannick

Belvis Nieto Robert

Ben Assayag Einor

Beniczky Sándor

Benussi Alberto

Bereczki Dániel

Berek Klaus

Berezowski Vincent

Berger Benjamin

Bersano Anna

Berthier Marcelo

Bertola Laiss

Berzero Giulia

Beudel Martijn

Bezdicek Ondrej

Bezzini Daiana

Bhaskar Sonu

Bhattacharyya S.

Bigi Sandra

Biscetti Leonardo

Bisdorff Alexander*

Bisecco Alvino

Bjørnevik Kjetil

Bø Lars

Bode Felix

Boentert Matthias

Bogovic Petra

Bohnen Nicolaas

Boller Francois

Bologna Matteo

Borges De Lacerda Glenda

Bornstein Natan

Boterberg Tom

Bottini Gabriella

Bougea Anastasia

Bouhassira Didier

Boychev Nikolay

Brandt Christian

Breiner Ari

Brigo Francesco

Brink‐Kjær Andreas

Brissette Vincent

Brochet Bruno

Brody Ethan

Broessner Gregor

Brownlee Wallace

Bruna J.

Brusch Anna

Bsteh Gabriel

Bu Bitao

Budhram Adrian

Bunn Lisa

Burke Matthew

Burst Volker

Burt R. K.

Burtscher Johannes

Butzkueven Helmut

Cagnan Hayriye

Cagnin Annachiara

Cakmak Elif Tarihci

Calandra‐Buonaura Giovanna

Calvo Andrea

Camargo Carlos Henrique

Camilo Mr

Cao Bei

Cao Bo

Caparros François

Caramelli Paulo

Carcel Cheryl

Carecchio M.

Carrette Evelien

Carroll Ian

Cartwright Michael

Carvalho De Oliveira Lara

Carvalho Vanessa

Casolla Barbara

Castrillo Sergio Muniz

Castrioto Anna

Caughey Byron

Cavaco Sara

Cavallieri Francesco

Cavanna Andrea Eugenio

Celius Elisabeth

Centanni Marco

Centen Liesanne

Cerami Chiara

Cerasa Antonio

Cereda Carlo

Chakravarty Ambar

Chamorro Angel

Chang Jason

Charlet‐Berguerand Nicolas

Chassain Carine

Chataway Jeremy

Chen Huisheng

Chen Jing‐Zhou

Chen Liang‐Kung

Chen Wenan

Chen Zhiyong

Chin Richard

Chiò Adriano

Chitnis Tanuja

Chitu Violeta

Choi Jay Chol

Choi Young‐Ah

Chollet François

Chong Trevor

Chu Kon

Chung Jinyoung

Cichosz Simon Lebech

Cimflova Petra

Ciucci Michelle

Clarke Robert

Clave Pere

Cock Hannah

Coebergh Jan

Colebatch James

Colonna Isabella

Comabella Lopez Manuel

Conceição Isabel

Consonni M.

Coon Elizabeth

Coote Skye

Coppola Nicola

Corboy John R.

Corcia Philippe

Cornejo‐Olivas Mario

Cornett Kayla

Correale Jorge

Cortese Andrea

Costa João

Coulson Elizabeth

Coutinho Jonathan

Covey Thomas

Cras Patrick

Crowe Byron

Cuadrado Elisa

Cubo Esther

Cupido Arjen

Cupler Edward

Cutsforth‐Gregory Jeremy

Czlonkowska Anna

Devos David

D'amico Emanuele

Daghlas Iyas

Dahodwala Nabila

Dal‐Bianco Assunta

Dalton Jarrod

Damato Valentina

D'ambrosio Alessandro

Dang Utkarsh

Dantonio Fabrizia

Dawson Deirdre

De Abajo Francisco José

De Camp Nora Vanessa

De Corte Thomas

De Herdt Veerle

De Marchi Fabiola

De Marchis Gian

De Mendonça Alexandre

De Meo Ermelinda

De Visser Marianne**

Deconinck Nicolas

Degirmenci Yildiz

Deiva Kumaran

Dekker Marieke

Del Felice Alessandra

Deli Tamas

Delmont Emilien

Deluca J.

Demetriades Andreas

Demeyer Simon

Deng Han Xiang

Deplanque Dominique

Derex Laurent

Dersch Rick

Desguerre Isabelle

Detke Holland C.

Deuschl Guenther

Devigili Grazia

Dhana Anisa

Di Cristofori Andrea

Di Fonzo Alessio

Di Lorenzo Francesco

Di Luca Monica

Di Paolo Therese

Di Piero Vittorio

Diamanti Susanna

Diegoli Henrique

Dietmann Anelia

Di Francesco Jacopo C.

Digirolamo Alessia

Digre Kathleen B.

Ding Jiayue

Ding Y. H.

Dinoto Alessandro

Diomedi Marina

Disanto Giulio

Divani Afshin

Dixon Luke

Dobler Gerhard

Donadio Vincenzo

Donaghy P.

Doneddu Pietro Emiliano

Doppler Kathrin

Dove Abigail

Doyle Karen

Drenthen G.

D'souza Marcus

Dua Kamal

Dubenko Andriy

Dubey Divyanshu

Ducros Anne

Dumitrascu Oana

Dupont Sophie

Durfee Alexandra

Durham Benjamin

Dusenbury Wendy

Dziedzic Tomasz

Eadie Mervyn

Ebinger Martin

Echaniz‐Laguna Andoni

Edwards Mark

Ehler Edvard

Elafros Melissa

Elangovan Cheran

Eling Paul

Elkind Mitchell

Eltoft Agnethe

Emile Jean‐Francois

Ercoli Tommaso

Erga Aleksander

Erhart Philipp

Erken Neziha

Espay Alberto

Esposito Fabrizio

Etminan Mahyar

Evangelopoulos Maria Eleftheria

Evers Stefan

Evoli Amelia

Ezekiel Leisle

Fabbri Margherita

Falup‐Pecurariu Cristian

Fanciulli Alessandra

Fandler‐Höfler Simon

Fanfulla Francesco

Farah Rita

Farhad Khosro

Farina Antonio

Fasano Alfonso

Ferini Strambi Luigi

Ferraro Diana

Ferro José

Festari Cristina

Filippakopoulou Eugenia

Filla Alessandro

Filosto Massimiliano

Fink Katarina

Finke Carsten

Finsterer Josef

Fiorio Mirta

Firbank Michael

Flanagan E. P.

Flemming Kelly D.

Flumignan Ronald

Foley Jennifer A.

Fonseca A. C.*

Fonseca Vivian

Ford Nathan

Foschi Matteo

Foster Erin

Foster Paul

Franz David N.

Freedman Mark S.

Freund Patrick

Frey Anna

Frisullo Giovanni

Fronczek R.

Frösen Juhana

Fujiwara Shunrou

Fung Victor

Gadaleta Giulio

Gaetani Lorenzo

Gaggioni Giulia

Gaig Carles

Galan Lucia

Galioto Rachel

Galvao‐Carmona Alejandro

Garavaglia Barbara

García Azorín David

Garcia Adolfo M.

García‐Azorín David

García‐Merino J. Antonio

Garcia‐Monco Juan Carlos

Garcia‐Ruiz Pedro

Gardiner Dale

Garg Ravindra Kumar

Garibaldi Matteo

Gärtner Jutta

Gatti Laura

Gdovinova Zuzana

Geppetti P.

Ghanbarnia Mohammad Javad

Ghozy Sherief

Giaccone Giorgio

Giannopoulos Sotirios

Gijs Marlies

Gilbert Donald

Gil‐Gouveia Raquel

Gilhus Ne

Gillespie David

Gilligan Michael

Giorgi Filippo

Goadsby Peter J.

Godefroy V.

Goedee H. Stephan*

Goetzl Laura

Gold Daniel

Goldstein D. S.

Gon Yasufumi

Gordish‐Dressman Heather

Görg Boris

Gorman Kathleen M.

Govaarts Rosanne

Goyal Vinay

Grandis Marina

Grigoriadis Nikolaos

Gromisch Elizabeth

Grosu Oxana

Grün Daniel

Grundtvig Josefine Liv Gilling

Guedes Bruno

Guekht Alla

Guenego Adrien

Guggisberg Adrian

Guglielmetti Monica

Guler Ayse

Gunduz Aysegul

Günther René

Guo Lei

Gustafsson Rasmus

Gwathmey Kelly G.

Habek Mario

Habib Ali

Haddad Naim Iskandar

Hagberg Katrina

Hagenacker Tim

Hahn Katrin

Haider Lukas

Hakim Alan J.

Hamilton R.

Hammerbeck Ulrike

Hammill Adrienne

Hancock Laura

Hansen Jakob Moller

Hao Zilong

Harbo Thomas

Hardy Todd A.

Haroche Julien

Harun Sabariah Noor

Harvey Calum

Haugarvoll Kristoffer

Havrankova Petra

Hayashi Yukiko

Helbok Raimund

Heldner Mirjam

Hellwig Kerstin

Hemelsoet Dimitri

Hemmer Bernhard

Hendriks Eef

Heo Ji Hoe

Herdman David

Hilz Max

Hinduja Archana

Hiramatsu Masafumi

Ho Roger

Hobson Peter

Hoepner Robert

Hoeritzauer Ingrid

Hoffmann Sarah*

Holmøy Trygve

Hong Daojun

Hong Zhen

Honig Asaf

Horisawa Shiro

Horvath Rita*

Hostettler Isabel

Houwen Saskia

Hugelshofer Michael

Huizinga Ruth

Huppert Doreen

Hvid Lars

Hwu Wuh‐Liang

Iacono Salvatore

Ichijo Hiroaki

Ihara Masafumi

Ihle‐Hansen Hakon

Iizuka T.

Iizuka Tomomichi

Ilinca Andreea

Infante Jon

Inglese Matilde

Irani Sarosh

Ishikawa Hidehiro

Islam Zhahirul

Izumi Yuishin

Jabbarli Ramazan

Jacob Saiju

Jacobs Bart*

Jaekel Anke

Jahanshad Neda

Jakel Lieke

Janbek Janet

Jankovic Milena

Jansa Jelka

Jarnes Jeanine

Jasne Adam

Jaunmuktane Zane

Ječmenica‐Lukić Milica

Jellinger Kurt

Jensen‐Kondering Ulf

Jia Min

Jiang Hong

Jin Huijuan

Jinnah Ha

Johannes Pfaff

Johansen Michelle

Johnson Karin

Jones Jacob D.

Jones Kelvin

Jongen Joost

Joubert Bastien

Joutsa Juho

Judith Wagner

Juega Jesus

Jurynczyk Maciej

Kachaner Alexandra

Kaesmacher Johannes

Kafaie Jafar

Kagerbauer Simone

Kalaoja Marita

Kalecký Karel

Kalincik Tomas

Kalinowska‐Lyszczarz Alicja

Kalita Jayantee

Kalla Roger

Kamouchi M.

Kan Hermien E.

Karaa Amel

Karam Chafic

Karavasilis Efstratios

Karenberg Axel

Kargiotis Odysseas

Karnon Jon

Kaski Diego

Katisko Kasper

Katzberg Hans

Kaufmann Horacio

Kearney Hugh

Kemchoknatee Parinee

Khalil Michael

Khatami Ramin

Khosdelazad Sara

Kiechl Stefan

Killestein Joep

Kim Beom Joon

Kim Byung Moon

Kim Byung‐Kun

Kim Gyeong‐Moon

Kim Ji‐Soo

Kim Jong

Kim Jong‐Min

Kim Joon‐Tae

Kim Su‐Hyun

Kim Yerim

Kimura Akio

Kister Ilya

Kitley Joanna

Kjaergaard Alisa

Kleine Justus F.

Kleinschnitz Christoph

Kleiter Ingo

Klimiec‐Moskal Elzbieta

Klimkowicz‐Mrowiec Aleksandra

Klinke Marianne Elisabeth

Klistorner Alexander

Kobayashi Ryota

Koehler Peter

Koga Masatoshi

Koike Haruki*

Kolmos Mia

Konen Franz

König Marton

Korsbæk Johanne

Kõrv Janika

Kossorotoff Manoelle

Kozberg Mariel

Krajnc Nik

Krarup Christian

Kremer Christine

Kreydin Evgeniy I.

Krishnan Arun V.

Kruer Michael

Kruijshaar Michelle

Kular Lara

Kusunoki Susumu

Kutlubaev Mansur

Kuwabara Satoshi*

Kwan Park

Kytövuori Laura

L. Abraira

La Bella Vincenzo

La Piana Roberta

Laakso Sini M.

Laffan Aoife

Laforce Jr. Robert

Lainez Miguel

Lambea‐Gil Álvaro

Lampl Christian

Landi Doriana

Lanfranconi Silvia

Lanteri‐Minet M.

Lanzino Giuseppe

Lanzone Jacopo

Larner Andrew

Lattanzi Simona

Laura Matilde

Laureys Guy

Le Panse Rozen

Lebedeva Elena

Lebrun‐Frenay Christine

Lechien Jerome

Leckman James

Lee J.

Lee Jay

Lee Yi‐Chu

Leger J. M.

Lehky Tanya

Lehmann Helmar

Lehmann Sylvain

Lehrer H. Matthew

Leinonen Ville

Leker Ronen

Lemmens Robin

Lemos Carolina

Leocani Letizia

Leta Valentina

Levy G.

Lewis Steven

Leypoldt F.

Li Qi

Li Yang

Li Yingkai

Lian Xuegan

Liebler Eric

Lim Ming

Lima Cesar

Lin Chen

Lin Hsien‐Ho

Lin Jie

Lindberg Påvel

Lindgren Arne

Lindsberg Perttu

Linnebank Michael

Lioutas Vasileios‐Arsenios

Lip Gregory

Liu Chenchen

Liu Fangzhou

Liu Jie

Liu Jun

Liu Ling

Lo Coco Daniele

Lockshin Md

Logroscino Giancarlo

Long Youming

Lootens Thibault

Lopes Carolina

Lopes Renaud

Louapre Céline

Louis Elan

Lövblad Karl‐Olof

Lozeron Pierre

Luca Antonina

Luis Guerrero‐Peral Ángel

Lun Ronda

Luongo Livio

Lv Ming

Lv Renjun

Lynch David S.

Ma Lu

Ma Yilong

Maarbjerg Stine

Maas Roderick

Macaron Gabrielle

Maccotta Luigi

Macerollo Antonella

Machado Calixto

Macleod Angus

Maclver Claire

Madjidyar Jawid

Madsen Tracy

Maestrini Ilaria

Maggi Lorenzo

Magy Laurent*

Magyari Melinda

Mahlknecht Philipp

Makita Naoki

Malfatti Edoardo

Malik Rayaz

Malkani Roneil

Mallela Deepthi Priyanka

Malm Jan

Malpas Charles

Malter Michael

Mammen Andrew L.

Man Shumei

Mancardi Giovanni Luigi

Mancuso Michelangelo

Manera Umberto

Manganelli Fiore

Manini Arianna

Mantegazza Renato

Mantokoudis Georgios

Marelli Sara

Mariotto Sara

Marriott James

Marsili Luca

Marson Anthony

Martelletti Paolo

Martial Charlotte

Martinelli Boneschi Filippo

Martinez‐Majander Nicolas

Martino Davide

Mas Jean‐Louis

Masdeu Joseph

Masson Catherine

Mastaglia F.

Matà Sabrina

Mathon Bertrand

Matías‐Guiu Jordi A.

Matsuoka Teruyuki

Matz Karl

Mayer Geert

Mayer‐Suess Lukas

Mazidi Mohsen

Mazzacane Federico

Mc Carthy Christine Eileen

Mccombe Pamela A.

Mcdonnell Katherine

Mcdonough Rosalie

Mckeon Andrew

Mckinley Richard Ian

Mecocci Patrizia

Mehndiratta Man Mohan

Mehta Rupal

Meisel Andreas

Meisinger Christa

Melzer Nico

Membrilla Javier

Menon Deepak

Menzler Katja

Meola Giovanni

Merlino Giovanni

Merten Natascha

Messinis L.

Meyer Jeffrey H.

Meyer Thomas

Meyts Isabelle

Micoulaud‐Franchi Jean‐Arthur

Midgley Adrian

Migliaccio Raffaella

Millecamps Stephanie

Miller Thomas

Minchell Ellie

Minor Philip

Mirmosayyeb Omid

Mishra Nishant

Misra Usha

Mistry Eva A.

Mitarnun Witoon

Mitsikostas Dimos

Miwa Kaori

Mizukami Hiroki

Mizuno Toshiki

Moccia Marcello

Mohamed Ibrahim Norlinah

Möhn Nora

Moisset Xavier

Mollan Susan

Mongin Alexander

Mongini Tiziana

Monica Margoni

Moniuszko Anna

Monschein Tobias

Montaner Joan

Montellano Felipe A.

Montvilas Erisela

Morales‐Briceno H.

Morbelli Silvia Daniela

Morgante Francesca

Mori Masahiro

Moriya Toshifumi

Moroni Isabella

Morotti Andrea*

Morris‐Bankole Hannah

Morrison Cassandra

Motyl Jiri

Moulin Thierry

Mueller Karsten

Muir Keith W.

Müller Jannis

Müller Roy

Munoz Luis

Muñoz‐Delgado Laura

Muraro Paolo

Murthy Jaya

Mychasiuk Richelle

Myslimi Fjorda

Nadareishvili Zurab

Naddaf Elie

Nadjar Yann

Næss Halvor

Nagel Simon

Naismith Robert T.

Nakagawa Masanori

Nakajima Madoka

Nakamizo Tomoki

Nanetti Lorenzo

Nascimento Osvaldo

Nasr Nathalie

Nassogne Marie‐Cécile

Navarro Pérez María Pilar

Navi Babak Benjamin

Neeb Lars

Nelson Kevin R.

Nemoto Kiyotaka

Neuhaus Oliver

Neumann‐Haefelin Christoph

Neuteboom Rinze

Ng Felix C.

Ngo Shyuan

Nguyen Thanh N.

Ni Jun

Nicita Francesco

Nicolas Gaspard

Nicoletti Alessandra

Nielsen Glenn

Niibo Takeya

Nishino Ichizo

Nobile‐Orazio Eduardo

Nocentini Ugo

Nordberg Agneta

Noubiap Jean Jacques

Nouri Aria

Novotny Vojtech

Noyce Alastair

Ntaios George

Nutma Erik

Nyquist Paul

Oaklander Anne Louise

O'connor Margaret

Ohashi Kazuki

Oishi N.

Okamoto Luis E.

Olafson Emily

Olaiya Muideen

Oliveira Fabricio

Oliver David

Ombrello Amanda

Onofrj Marco

Oostema John Adam

Orngreen Mette

Ørstavik Kristin

Orsucci Daniele

Orthmann‐Murphy Jennifer

Otite Fadar Oliver

Outteryck Olivier

Owolabi Mayowa

Paddick Stella‐Maria

Pajediene Evelina

Pal Gian

Palaiodimou Lina

Palazzo Paola

Palmini Andre

Pana Tiberiu

Pandey Sanjay

Pantoni Leonardo

Papadaki Efrosini

Papadopoulos Constantinos

Papagianni Aikaterini

Papagno Costanza

Papanagiotou Panagiotis

Papier Paulina

Parchi Piero

Parees Isabel

Pareyson Davide*

Parikh Neal

Park Hyung Jong

Parman Yesim

Parnetti Lucilla*

Parson Simon H.

Parsons Mark

Pascual Julio

Pasi Marco

Pastor P.

Patejdl Robert

Paul Friedemann

Pavăl Denis

Pavlovic Aleksandra

Pearlman Daniel M.

Pelosi Luciana

Pelz Johann

Penner Iris‐Katharina

Pennuto Maria

Perani Daniela

Pereira Cristiana

Perepezko Kate

Perez David L.

Perez‐Lloret Santiago

Perić Stojan*

Pernet Vincent

Peters N.

Petersen Nils H.

Petri Susanne*

Petropoulos Ioannis

Petrulis Mary

Petzold Axel

Pfausler Bettina

Picillo Marina

Pico F.

Piehl Fredrik

Pilato Fabio

Pilotto Andrea

Pinho João

Pinto Susana

Pitarokoili Kalliopi

Plantone Domenico

Plummer Prudence

Poesen Koen

Poisnel Geraldine

Ponchelle‐Dequatre Nelly

Pontieri Francesco

Poos Jm

Popp Julius

Postuma Ronald B.

Pot Caroline

Pradat Pierre‐François

Prats‐Sánchez Luis

Premi Enrico

Previtali Stefano Carlo*

Primiano Guido

Pringsheim Tamara

Prosperini Luca

Ptak Radek

Pul Refik

Putaala Jukka

Puy Laurent

Qiu Chengxuan

Qiu Wei

Radojewski Piotr

Radovanovic Sasa

Raffaelli Bianca

Raggi Alberto

Rahouma Mohamed

Raja Shruti

Rajabally Yusuf A.

Ramanathan Sudarshini

Ramdharry Gita

Ramirez‐Zamora Adolfo

Ransohoff Richard R.

Ranta Anna

Rao Renata

Rattay Tim

Reddel Stephen

Refaee Ehab El

Refardt Julie

Reilly Mary*

Rejdak Konrad

Rektor Ivan

Rektorova Irena

Religa D.

Reuter Uwe

Revuelta Gonzalo

Rex Nathaniel

Rheims Sylvain

Richard Sébastien

Rimkus Carolina

Rimmele David

Rinaldi Carlo

Ripellino Paolo

Ristic Aleksandar

Riva Nilo

Roberts Nicole

Rocca Maria

Rodriguez‐Oroz María Cruz

Rodríguez‐Violante Mayela

Romano Marcello

Romito Luigi

Ronchi Dario

Rosner Jan

Rossetti Andrea

Rössler Karl

Rossor Alex

Rostasy Kevin

Roth Patrick

Rovira Alex

Roze Emmanuel

Rubegni Anna

Rudilosso Salvatore

Rudnik‐Schoeneborn Sabine

Rüegg Stephan

Ruigrok Y. M.

Russell Lucy

Rzepiński Łukasz

S. Shripad Pujari

Sabatelli Mario

Sabater Lidia

Saccà Francesco

Saha Ram

Saidha S.

Saigoh Kazumasa

Sairanen Tiina

Sakthivadivel Varatharajan

Salerno Alexander

Salinas Meagen

Salmaggi A.

Salmen Anke

Salom Juan

Salter Amber

Sander Josemir

Sandor Cynthia

Sangalli Davide

Sani Ilaria

Sanjuan Estela

Sansone Valeria

Santalucia Paola

Santamarina Estevo

Santangelo Gabriella

Santillo Alexander

Santorelli Filippo M.

Sara Cabras

Sarasate Mikel

Saro‐Buendía Miguel

Sarva Harini

Sastre‐Garriga Jaume

Saylor D.

Scheffel Lucia

Scheitz Jan

Schellinger Peter

Schenck C. H.

Schiava Marinela

Schiavetti Irene

Schievink Wouter I.

Schlegel Uwe

Schlenstedt Christian

Schmidt Jens

Schmtt Oliver

Schmutzhard Erich

Schneider R.

Schneider Susanne A.

Schneider‐Gold Christiane

Schoenen Jean

Schoonheim Menno

Schoser Benedikt

Schrag Anette

Schreiber Herbert

Schwab Nicholas

Schwingenschuh P.

Scivoletto G.

Scutelnic Adrian

Sechi Elia

Sedova Petra

Seeman Pavel

Seghier Mohamed

Seiffge David

Seiler Andrea

Sejbaek Tobias

Sejersen Thomas

Sekula Raymond F.

Sellner Johann*

Selvarajah Dinesh

Sen Souvik

Senderek Jan

Seners Pierre

Serranova Tereza

Sessa Francesco

Shafer Jennifer S.

Shahrizaila Nortina*

Shefner Jeremy

Shekeeb Mohammad

Shemie Sam

Shen Ying

Sheng Can

Shepherd Claire

Shi Yachen

Shimizu T.

Shimizu Yuko

Shimoyama Takashi

Shin Hyeong‐Geol

Sidiropoulos Christos

Siegler James

Siepmann Timo

Signori Alessio

Sigurdsson Hilmar

Silani Vincenzo

Silberstein Stephen

Silverdale Monty

Silveri Maria Caterina

Silvestri Gabriella

Singer Madeline

Sinha Sanjay

Skipper Caleb P.

Skogseid Inger Marie

Skorvanek Matej

Smeralda Carmelo

Smith Eric

Smyth Duncan

Snowden John A.

Sobczyk Maria K.

Soffietti Riccardo

Solomon Andrew

Song Bo

Soni R.

Sonoo Masahiro

Sorarù Gianni

Sousa Mário

Souter Michael

Spatola Marianna

Spence J. David

Spina Alfio

Sposato Luciano

Stark Richard

Staub Daniel

Staykov Dimitre

Stefani Ambra

Steinhoff Bernhard

Stevanin Giovanni

Stojkovic Tanya

Stoll Guido

Stone John

Strle Franc

Strzelczyk Adam

Suller Marti Ana

Summ Oliver

Sumner Petroc

Sun Haitao

Sun Ji

Sunara Ivan

Sun‐Edelstein Christina

Suzuki Keisuke*

Suzuki Naoki

Suzuki Tomoaki

Swart Grace

Taba Pille

Tábuas‐Pereira Miguel

Takeda Takahiro

Takizawa Tsubasa

Tamburin Stefano

Tan Eng‐King

Tan Xiaoping

Tanasescu Radu

Tankisi Hatice

Tant Mark

Tarkka Ina

Tarnutzer Alexander A.*

Tateno Amane

Taubøll Erik

Taylor Bruce V.

Tecchio Franca

Teive Helio

Tenberg Sarah

Teodoro Tiago

Ter Schiphorst Adrien

Terman Sw

Terzi Murat

Tessitore Alessandro

Theaudin Marie*

Thijs Roland

Thijs Vincent N.

Thom Maria

Thomas Aline

Thome Claudius

Ticozzi Nicola

Tienari Pentti

Tigano Marco

Timmerman Hans

Timsit Serge

Titcomb Tyler*

Tobis Jonathan M.

Todd Candice

Toft Mathias

Tolle Virginie

Tomaselli Pedro José

Tomic Svletana

Topcuoglu M.

Topiwala Anya

Torricelli Ricardo

Tortuyaux Romain

Tourdias Thomas

Toyoda Hidenori

Traenka Christopher

Tranchant Christine

Trevisi Gianluca

Tripoliti Elina

Trojsi Francesca***

Truini Andrea

Tsivgoulis Georgios*

Tsolaki Magda

Tsuji Shoji

Turner Clinton P.

Tutino Vincent

Tuttolomondo Antonino

Tuzun Erdem

Uchiyama Sninichiro

Ueda Mitsuharu

Ueno Yuji

Uher Tomas

Umapathi T.

Uncini Antonino

Urra Xabier

Urso Daniele

Uyttenboogaart Maarten

Uzawa Akiyuki

Vakulin Andrew

Vale Fernando L.

Van Amerongen Suzan

Van Damme Philip

Van De Munckhof Anita

Van Der Beek Nadine

Van Der Meijden Chris

Van Dijk J. Gert

Van Doorn Pieter A.

Van Eijk Jeroen

Van Gool Wa

Van Schependom Jeroen

Van Wamelen Daniel

Vandenberghe R.

Vanoli Fiammetta

Varkanitsa Maria

Vecchié Alessandra

Veje Malin

Velasco Roser

Vencovsky J.

Verde Federico

Verger Antoine

Vergouwen Mervyn

Vermersch Patrick

Vernieri Fabrizio

Verschuuren Jan

Vethanayagam Dilini

Viader Fausto

Vibha Deepti

Vibo Riina

Vidailhet Marie

Vieira Helena L. A.

Vijayan Joy

Vissing John

Vitturi Bruno

Vodusek David B.

Vollert Jan

Von Oertzen Tim

Von Wyl Viktor

Voss Joachim

Voysey Zanna

Vriend Chris

Vucic Steve

Vukusic Sandra

Wada Kanichiro

Waheed Waqar

Waje‐Andreassen Ulrike

Waliszewska‐Prosol Marta

Walter Silke

Wang Hao‐Kuang

Wang Junhui

Wang Lin

Wang Zeneng

Warnke Clemens

Warren Nicola

Watanabe Hirohisa

Weiler Markus

Weiss Miriam

Wellwood Ian

Wenninger Stephan

Werring David

Weydt Patrick

White Andrew J.

Whitehouse William

Wiendl Heinz

Wieske Luuk

Willekens Barbara

Willemse Eline

Winkler Juergen

Wirdefeldt Karin

Witzle Simon

Woldeamanuel Yohannes Woubishet

Wolfe Charles

Wolke Robin

Wong George K. C.

Wrona Pawel

Wu Ruey‐Meei

Wu Teddy Y.

Wu Zhi‐Ying

Xia Fan

Xiao Jiayu

Xing Yan

Xiong Li

Xu Gelin

Xu Ying

Xue Qianli

Yabe I.

Yadav Ravi

Yaguchi Hiroaki

Yakushiji Yusuke

Yamazaki Yu

Yang Li

Yang Xiaorong

Yau Wai Yan

Yener Görsev

Yesilot Nilufer

Yildiz Yilmaz

Yoon Woong

Yoshimoto Takeshi

Yuan Zengqiang

Zakaria Magd

Zandi Michael S.

Zanusso Gianluigi

Zecca Chiara

Zedde Marialuisa*

Zedde Ml

Zekerdiou Anastasia

Zelano Johan

Zeng Jinsheng

Zerr Inga

Zeuner Kirsten

Zhang Guoqiang

Zhang Hong‐Liang

Zhang Zaiqiang

Zhao Guohua

Zhao Xingquan

Zheng Ming

Zhou Helen

Zhou Hongyu

Zhou Junhong

Zhou Lily

Zhu Chengcheng

Zhu Yi‐Cheng

Ziemssen Tjalf

Zimprich Fritz

Zini Andrea

Zis Panagiotis

